# Altered corpus callosum structure in adolescents with cerebral palsy: connection to gait and balance

**DOI:** 10.1007/s00429-023-02692-1

**Published:** 2023-08-24

**Authors:** Julia Jaatela, Timo Nurmi, Jaakko Vallinoja, Helena Mäenpää, Viljami Sairanen, Harri Piitulainen

**Affiliations:** 1 Department of Neuroscience and Biomedical Engineering, Aalto University School of Science, 02150 Espoo, Finland; 2 Faculty of Sport and Health Sciences, University of Jyväskylä, 40014 Jyväskylä, Finland; 3 Department of Neurology, New Children’s Hospital, Helsinki University Central Hospital, 00029 Helsinki, Finland; 4 Department of Clinical Neurophysiology, BABA Center, Pediatric Research Center, Children’s Hospital and HUS Imaging, Helsinki University Central Hospital, 00029 Helsinki, Finland; 5 Department of Radiology, Kanta-Häme Central Hospital, 13530 Hämeenlinna, Finland; 6 Aalto NeuroImaging, Aalto University, 02150 Espoo, Finland

**Keywords:** Diffusion-weighted MRI, Tractography, Stability, Transcallosal, Interhemispheric

## Abstract

Cerebral palsy (CP) is the most common motor disorder in childhood. Recent studies in children with CP have associated weakened sensorimotor performance with impairments in the major brain white-matter (WM) structure, corpus callosum (CC). However, the relationship between CC structure and lower extremity performance, specifically gait and balance, remains unknown. This study investigated the transcallosal WM structure and lower limb motor stability performance in adolescents aged 10–18 years with spastic hemiplegic (*n* = 18) or diplegic (*n* = 13) CP and in their age-matched controls (*n* = 34). The modern diffusion-weighted MRI analysis included the diffusivity properties of seven CC subparts and the transcallosal lower limb sensorimotor tract of the dominant hemisphere. Children with CP had comprehensive impairments in the cross-sectional area, fractional anisotropy, and mean diffusivity of the CC and sensorimotor tract. Additionally, the extent of WM alterations varied between hemiplegic and diplegic subgroups, which was seen especially in the fractional anisotropy values along the sensorimotor tract. The diffusion properties of transcallosal WM were further associated with static stability in all groups, and with dynamic stability in healthy controls. Our novel results clarify the mechanistic role of the corpus callosum in adolescents with and without CP offering valuable insight into the complex interplay between the brain’s WM organization and motor performance. A better understanding of the brain basis of weakened stability performance could, in addition, improve the specificity of clinical diagnosis and targeted rehabilitation in CP.

**Supplementary Information:**

The online version contains supplementary material available at 10.1007/s00429-023-02692-1.

## Introduction

Cerebral palsy (CP) is a permanent movement and posture disorder caused by a defect or lesion in the developing brain (Bax et al. 2005). The most common clinical feature is spasticity, typically affecting the body bilaterally (diplegia) or predominantly unilaterally (hemiplegia). Despite the wide heterogeneity of CP, an increasing number of studies have shown that both hemiplegic and diplegic CP affect an individual’s gait and balance control (Wang and Wang 2012; Rojas et al. 2013; Bruijn et al. 2013; Pavão et al. 2015; Piitulainen et al. 2021). However, the importance of the structural deficits in the white matter (WM) of the brain for the impaired sensorimotor proficiency in CP remains poorly investigated.

Several studies have suggested that corpus callosum (CC), the major WM pathway connecting the two hemispheres, is one of the main brain structures affected in CP (for a review, see (Scheck et al. 2012). Children with CP have smaller CC volumes (Hayakawa et al. 1996; Kułak et al. 2007; Hawe et al. 2013) and the WM structure in CC is altered (Weinstein et al. 2014; Arrigoni et al. 2016). Typically, the structural impairments have been studied in the whole CC or separately in its genu, body and splenium (Hayakawa et al. 1996; Kułak et al. 2007; Reid et al. 2015; Pagnozzi et al. 2020). The development of diffusion-weighted MRI (dMRI) and tractography methods have allowed for a more fine-grained approach to studying the WM connections between certain cortical areas (Arrigoni et al. 2016; Ballester-Plané et al. 2017; Papadelis et al. 2019; Mailleux et al. 2020). Although there is increasing knowledge about the involvement of CC deficits in CP, much uncertainty still exists about the extent of these deficits in the subtypes of CP, such as hemiplegia and diplegia.

The CC is considered important for the efficient sensorimotor function of the whole body. The structural properties of CC have been repeatedly associated with the bimanual performance both in CP (Weinstein et al. 2014; Hung et al. 2019; Robert et al. 2021) and in normal development (for a review, see Gooijers and Swinnen 2014). In addition, the severity of CP has been linked with the degree of the structural CC deficits: greater motor impairments according to the Gross Motor Functioning Classification Scale (GMFCS), have been associated with a smaller CC volume (Kułak et al. 2007; Reid et al. 2015) and decreased fractional anisotropy (FA) values in the CC (Lee et al. 2011; Ballester-Plané et al. 2017; Jiang et al. 2019). Nevertheless, only a few studies have attempted to link the CC anatomy to the lower extremity performance in CP. In spastic CP, worse gait performance has been associated with the lower volume of the total CC and its anterior part (Meyns et al. 2016) and with lesion locations in the anterior CC (Papageorgiou et al. 2020). Supporting evidence of CC involvement in dynamic balance has been shown in preterm born adolescents without CP (Groeschel et al. 2019). To extend the existing mechanistic understanding of motor impairments in CP, we aimed to investigate the role of the transcallosal WM connections in the sensorimotor function of the lower limbs.

Our primary aim was to compare the size and detailed WM structure of the CC between children with spastic hemiplegic or diplegic CP and their typically developed age-matched controls. We hypothesized that CP would be associated with a reduced area, and lower FA and higher mean diffusivity (MD) in the CC cross-section and, that these differences would be most prominent in the posterior subparts of the CC, following previous research (Lee et al. 2011; Scheck et al. 2012; Arrigoni et al. 2016). We further hypothesized that the structure of the transcallosal sensorimotor tract for the lower limb is altered in CP along its entire course. Our secondary aim was to examine whether the structural properties of the CC cross-section and the transcallosal sensorimotor foot tract are associated with the motor performance, more specifically with the static standing and dynamic gait stability. In a previous work of our research group, the static and dynamic stability measures were shown to differ between children with CP and controls (Piitulainen et al. 2021). We hypothesized that higher FA and lower MD, possibly reflecting more coherently organized WM connections, would be associated with better motor performance.

## Materials and methods

### Participants

#### Patients

This study included 31 children and adolescents aged 10–18 years with a confirmed diagnosis of spastic hemiplegic (HP: 13.4 ± 2.3 years, *n* = 18, 12 female) or diplegic (DP: 13.0 ± 2.1 years, *n* = 13, 6 female) CP. Their GMFCS level was I–II, meaning the ability to walk independently but limited to minimal ability to perform gross motor skills such as running and jumping (Palisano et al. 1997). Except for three CP participants with Full-Scale IQ (FSIQ) under 70, all were cognitively within normal variation (Wechsler Adult Intelligence Scale/Wechsler Intelligence Scale for Children; FSIQ score: 91.6 ± 18.3, range: 43–117; seven participants missing a test score). They had no known cognitive or co-operative deficiencies, hearing deficit, visual deficit other than refractive error, condition (other than CP) or medication known to affect gait and balance. Most of the DP participants were right-handed (9/13; mean score: 33.9, range: – 100 to 100) when assessed with the Edinburgh Handedness Inventory test (Oldfield 1971). For the hemiplegic participants, the dominant hand was defined as the less-affected hand (6/18 with dominant right hand).

#### Controls

34 Typically developed controls (TD: 13.9 ± 2.4 years, 20 female) volunteered in this study. They had no known neurological deficits or medication and they were all cognitively within normal variation (Wechsler Adult Intelligence Scale/Wechsler Intelligence Scale for Children; FSIQ score: 108.0 ± 14.9, range: 77–135; seven participants missing a test score). The majority of the TD participants were right-handed (32/34, Edinburgh Handedness Inventory mean score: 71.3, range: – 87 to 100, one participant missing a test score).

All participants were asked to attend both the stability assessment (static and dynamic stability) and the structural brain imaging. The two measurement sessions were conducted on separate days with a maximum of 2.5 months apart. Due to schedule challenges or inability to undergo an MRI study (discomfort or movement during the measurement or non-removable metal in the body), the number of participants in each study was the following: stability assessment 18 HP, 10 DP and 31 TD; and brain imaging 16 HP, 11 DP and 30 TD. See Supplementary Table S1 for detailed participant demographics. MRI images and CC cross-sections for participants with CP are presented in Supplementary Figure S1.

The study was approved by the Helsinki University Hospital ethics committee (HUS/2318/2016) and was in accordance with the recommendations of the Declaration of Helsinki. Informed written consent was obtained from all participants and their guardians before the experiment was conducted. The children with CP were recruited from the rehabilitation unit of Children´s Hospital, Helsinki University Hospital and by advertising via a patient organization. The healthy peers were recruited from school visits, the university and hospital staff’s family members and acquaintances.

### Static and dynamic stability testing

**Fig. 1:**
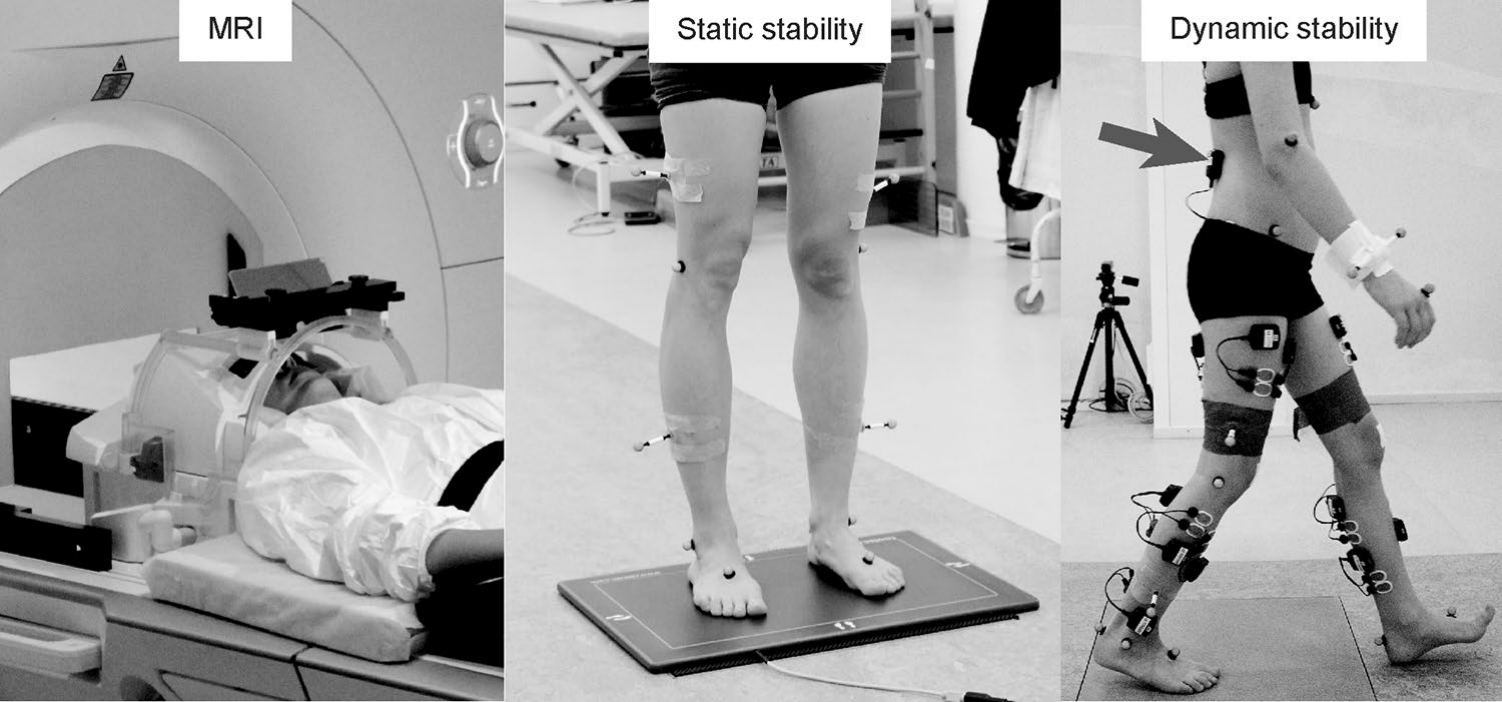
Experimental setups in MRI, static stability and dynamic stability measurements. The arrow indicates the location of inertial measurement unit on the lower back of the subject during dynamic gait stability testing

Static standing and dynamic gait stability were tested in the Motion Analysis Laboratory of the New Children’s Hospital, Helsinki University Hospital, Finland. Figure 1 illustrates the experimental setup. To assess the static standing stability, we recorded postural sway (center-of-force velocity, mm/s) using a plantar-pressure plate (0.5 m Hi-End Foot-scan® system, RSscan international, Brussels, Belgium) at 33 samples/s. Participants were instructed to stand as still as possible for 30 s while performing four separate tasks: (1) standing eyes open and feet pelvis-width apart (distance between anterior superior iliac spines), (2) standing eyes closed and feet pelvis-width apart, (3) standing with eyes open and feet together and (4) standing with eyes closed and feet together. The postural sway was quantified as average center-of-force velocity (mm/s) during each tasks separately.

Dynamic stability was quantified from body acceleration recorded with an inertial measurement unit (NGIMU, x-io Technologies Limited, Bristol, UK) during walking up-and-back a 10-m walkaway at a preferred speed. The unit was placed on mid-back at L3–L5 level with an elastic Velcro-strap. Three different tasks were implemented: (1) *normal* unconstrained gait, (2) *motor* dual task where the participant carried a tray with an empty mug on top of it, and (3) *cognitive* dual task where the participant listed aloud semantic (animals or foods and drinks) or phoneme (words starting with s or k) class words as fast as possible in accordance with the NEPSY-II assessment (Korkman et al. 2007). Each pass of the 10-m walkway cycle was identified, and initial and final portions of the gait were removed from the analysis. Refined-compound-multiscale entropy (https://github.com/tjrantal/javaMSE; Ihlen et al. 2016) indices were computed independently for vertical and resultant horizontal accelerations from each pass and averaged for the three tasks (for more details, see Piitulainen et al. 2021).

To assess the overall stability, the sum variables were constructed for the static and dynamic stability separately. Each measure was first normalized across the whole studied population to scale from 0 to 1 (from the best performance to the worst). Then, the test values were averaged for each participant to obtain the sum variable. The internal consistency of the sum variables items was estimated using Cronbach’s alphas and the values above 0.8 were considered to indicate good internal consistency (Cronbach 1951; Nunnally and Bernstein 1994).

### MRI data acquisition

MRI data collection using a 3T MAGNETOM Skyra MR scanner (Siemens Healthcare, Erlangen, Germany) with a 32-channel head coil is presented in Fig. 1. MRI measurements were performed at the Advanced Magnetic Imaging Centre of Aalto NeuroImaging. The imaging was performed without sedation or medication, while the participants were awake and relaxed.

The imaging protocol consisted of T1 and diffusion-weighted sequences. For structural images we used a T1-weighted magnetization-prepared rapid-acquisition gradient-echo (MPRAGE) sequence [voxel size = 1 mm^3^; field of view (FOV) = 256 × 256 mm; reconstructed matrix = 256 × 256; slices = 176; repetition time (TR)/echo time (TE) = 2.53 s/3.3 ms; flip angle = 7°]. Diffusion-weighted images were acquired using a single-shot EPI spin echo pulse sequence [voxel size = 2.5 mm^3^; FOV = 240 × 240 mm; reconstructed matrix = 96 × 96; slices = 70; TR/TE = 8.3 s/81 ms; flip angle = 90°]. For each participant, we measured 64 gradient directions with *b* = 1000 s/mm^2^ and 8 acquisitions with *b* = 0 s/mm^2^. Four *b* = 0 images were gathered in posterior–anterior and four in anterior–posterior phase encoding direction.

### MRI data processing

All MRI images were visually checked for any pronounced artefacts. Cortical reconstruction and volumetric segmentation of the T1-weighted structural images were performed using the Freesurfer image analysis suite (version 6.0-linux, http://surfer.nmr.mgh.harvard.edu; Dale et al. 1999; Fischl et al. 2002). The segmentation and the pial surface outline were manually checked and, if necessary, corrected. We observed that the automatic volumetric segmentation of CC was imprecise in the patient group and would have required extensive manual editing. To obtain a more rigorous segmentation, the CC was outlined manually at one sagittal slice at the midline, using the information of the fused dMRI and Freesurfer segmentation. This midsagittal CC area was then divided into seven geometrically defined subparts (Witelson 1989) using a custom script on Matlab (R2021b, Mathworks, Natick, Massachusetts, United States). Parcellation is presented in Fig. 3 top panel.

The dMRI data were first converted to 4D NIFTI files with dcm2niix (Li et al. 2016). Motion correction and eddy current corrections were executed using FMRIB’s Software Library tools eddy and topup (FSL version 6.0; Andersson et al. 2003; Smith et al. 2004). An estimate of the susceptibility-induced off-resonance field was obtained from the *b* = 0 images. All further processing of the dMRI data was conducted with the ExploreDTI software (version 4.8.6, https://www.exploredti.com; Leemans et al. 2009). Any remaining geometric deformations were removed using the T1 image as an undistorted reference, allowing non-linear deformations only along the anterior–posterior direction. At this step, the dMRI data were interpolated to a 1 mm^3^ voxel size, matching the T1 resolution. After the preprocessing of dMRI data, the outlier profiles and data quality were visually checked using the tools provided by ExploreDTI software.

First, we estimated voxel-wise diffusion tensors using the robust extraction of kurtosis indices with linear estimation algorithm (REKINDLE; Tax et al. 2015) that can detect and exclude movement-induced artefacts on the data. Second, to estimate fiber orientation distribution (FOD), we applied constrained spherical deconvolution (CSD) with 8th order spherical harmonics using recursive calibration of the response function (Tax et al. 2014). In contrast to tensor models, this method is capable of representing complex WM structures, e.g., crossing fibers.

For whole-brain fiber tracking, we used the deterministic algorithm (Jeurissen et al. 2011) implemented in ExploreDTI with the following parameters: seed point resolution = 1 mm, step size = 1 mm, angle threshold = 30, fiber length range = 20–250 mm and FOD threshold = 0.1. We then selected streamlines that originated from the sensorimotor cortical area (precentral gyrus, postcentral gyrus and paracentral lobule Freesurfer parcellations) and reached the midsagittal CC area. To achieve high robustness and consistency of the sensorimotor transcallosal tracts, we selected only the sensorimotor cortical area of the unaffected hemisphere for HP or the dominant hemisphere for TD and DP. Further, the Freesurfer cortical reconstruction was used to exclude fibers crossing any sulci. Finally, the generated streamlines were trimmed manually to include only fibers reaching the medial wall of the paracentral lobule or the most superior parts of the SMI gyrus, following the well-known homunculus of the lower limb (Penfield and Jasper 1954). The trimming thus resulted in fiber bundle with minimal fanning or branching and can be expected to represent the transcallosal sensorimotor tracts of the lower limb, also referred to as “transcallosal foot tracts”.

All analyses on MRI data were performed in individual anatomy. From the seven CC subparts, we extracted the cross-sectional area (mm^2^), average fractional anisotropy (FA) and mean diffusivity (MD). We found it important to report both the FA and MD values as they do not always change together (Yoshida et al. 2011). The CC area measurements were used as absolute values, given that we observed no statistically significant difference in the possible normalization factor, the intracranial volume, between the groups (Kruskal–Wallis, *p* = 0.084) and the normalized vs. un-normalized results appeared similar with visual inspection. The transcallosal fiber bundles were analyzed in three uniformly sampled sections along their trajectory between CC and cortex. These three sections were labelled as proximal (closest to the CC), middle (middle section) and distal (furthest from the CC, closest to the cortex). The FA and MD values were extracted for each tract section. Finally, we analyzed the average location of the transcallosal foot tracts on the CC subparts.

### Statistical analyses

Statistical analyses were performed with R statistical software (version 4.0.4; R Core Team 2020) To compare the demographic and clinical data between the CP and control groups, we applied the Kruskal–Wallis H test (Kruskal and Wallis 1952). In the case of statistically significant differences (*p* < 0.05), the Conover post hoc test was used to determine possible pair-wise differences (Conover 1999). Post hoc tests were FDR-corrected for multiple comparisons (Benjamini and Hochberg 1995).

For the structure−function analyses, we used two methods. First, we built a multiple linear regression model (lm function, R statistical software, Wilkinson and Rogers 1973; Chambers 1992) with dependent variables being the static and dynamic balance. The simultaneous independent variables were the size/FA/MD of the seven CC subparts, the FA/MD of the three transcallosal tract segments, and the group (HP, DP or TD), age and sex of the participants. Secondly, to study the group-wise correlations, we used the two-tailed Spearman correlation corrected for age (Kim 2015). To account for multiple comparisons, we applied the Bonferroni correction for each group separately.

## Results

Supplementary Table S1 lists the availability of data for each participant. Out of 65 participants, 4 did not attend any behavioral stability testing because of schedule challenges or other personal reasons. Additionally, the static stability was not measured on two DP participants, because the other had schedule challenges and the other could not perform the test because of the inability to stand still without support. One TD participant was excluded from the dynamic stability analysis because of technical connection problems in the wireless inertial measurements. The static (*n* = 59) and dynamic (*n* = 61) stability data included in the final analysis was of adequate quality.

We were unable to measure MRI for 8 participants due to schedule challenges (1 HP), anxiety towards MRI measurement (2 TD, 1 HP, 1 DP), metal in the body (1 TD, 1 DP) or excessive movement during measurement (1 TD). Furthermore, one HP and one DP participant participated only in structural T1-imaging, not dMRI. Based on visual inspection, the MRI data (T1 *n* = 57; dMRI *n* = 55) were of satisfactory quality and tractography was successful in all participants. Figure 4A–C show an example of the extracted sensorimotor transcallosal tracts and the analyzed tract segments in one representative control participant.

Importantly, no significant differences were found in age (*p* = 0.62) or gender (*p* = 0.52) between the three study groups.

### Children with CP show impaired standing and gait stability

The sum variables used to estimate the stability performances had a good consistency (Cronbach’s alpha 0.88 for static and 0.92 for dynamic stability items). Further, the two stability scores correlated strongly with each other (rho 0.56, *p* < 0.001).

**Fig. 2:**
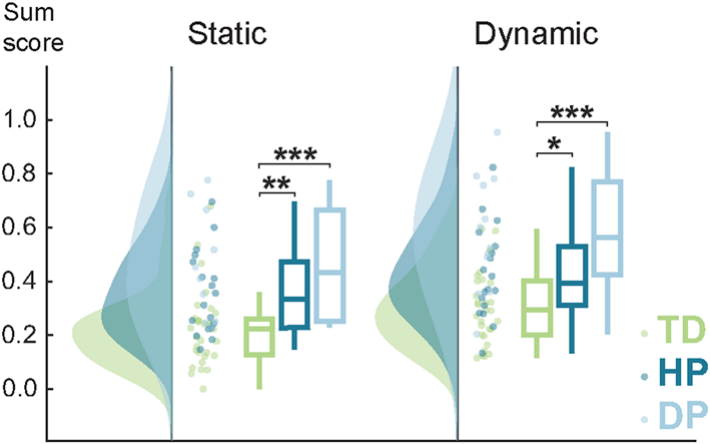
Static and dynamic stability. The sum stability scores differed significantly between children with CP (HP and DP) and controls (TD) but not between the two CP subgroups. Higher sum score indicates poorer stability performance. *p < 0.05, **p < 0.01, ***p < 0.001

Figure 2 shows the stability results for the three groups that differed significantly (*p* < 0.001) in both stability measures. Both CP subgroups showed significantly impaired stability performance, i.e. higher sum scores, in the static (*p* = 0.002 for HP and *p* < 0.001 for DP) and dynamic stability (*p* = 0.011 for HP and *p* < 0.001 for DP). Although children with DP appeared to have poorer stability performance than HP, no statistically significant differences were detected between the patient subgroups (*p* = 0.24 and *p* = 0.07 for the static and dynamic stability, respectively).

### Children with CP show structural impairments in the corpus callosum

**Fig. 3:**
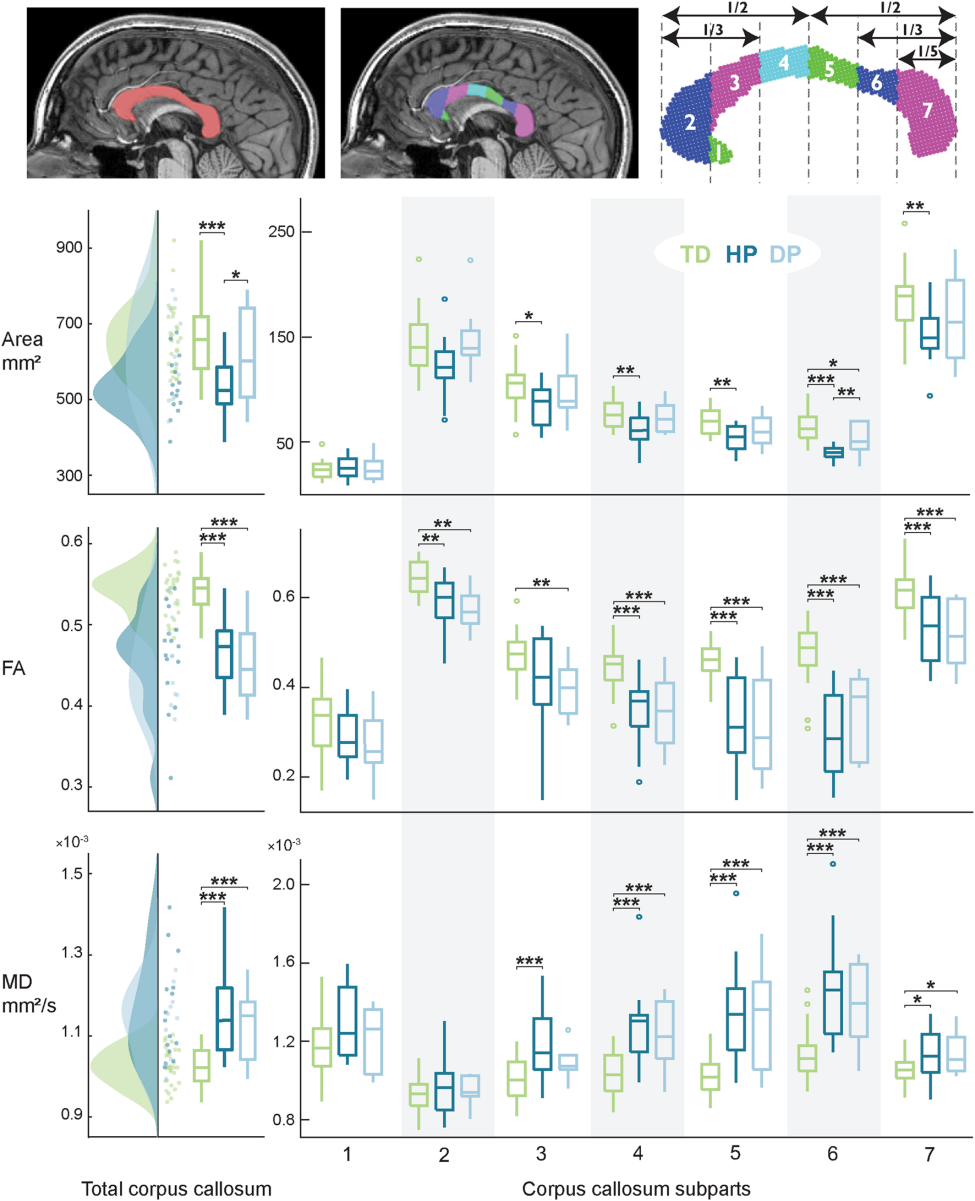
Cross-sectional area, average fractional anisotropy (FA) and mean diffusivity (MD) values of the total corpus callosum and its subparts. Statistically significant differences were observed in almost every subpart, and in each of the three metrics. *p < 0.05, **p < 0.01, ***p < 0.001

The size and diffusivity of the CC were reduced in CP. Figure 3 shows the group-wise differences in the area, average FA and average MD values for the total CC cross-section and for each of the seven CC subparts separately. The three groups differed statistically in all metrics and differences were visible in all CC subparts except in the rostrum (subpart 1). The majority of the statistically significant differences were observed between patients and controls, but some were observed also between HP and DP groups.

Children with hemiplegia had a reduced total CC area compared to both DP group (*p* = 0.032) and controls (*p* < 0.001). The HP group cross-sectional areas were smaller than TD across the body and splenium of the CC (*p* = 0.023 in subpart 3, *p* = 0.004 in 4, *p* = 0.003 in 5, *p* < 0.001 in 6 and *p* = 0.002 in 7). The isthmus (subpart 6) was significantly smaller for HP compared to DP group (*p* = 0.006). The isthmus area was also reduced for the DP group compared to controls (*p* = 0.020).

The two CP subgroups showed a greater degree of isotropic diffusion in the CC compared to controls. The FA values of the total CC were significantly reduced for both HP and DP (*p* < 0.001 for both groups). Similarly, HP group showed reduced FA values in subparts 2 (*p* = 0.002) and 4–7 (*p* < 0.001) and DP group in subparts 2–7 (*p* = 0.002 for subpart 2, *p* = 0.004 for 3, *p* < 0.001 for 4–7) compared to TD group. No statistically significant differences were detected between the two patient subgroups.

The average diffusivity in the total CC was significantly higher for both HP and DP groups (*p* < 0.001). Significantly higher MD values were observed across the body and splenium for HP (*p* < 0.001 in subparts 3–6, *p* = 0.050 in subpart 7) and DP (*p* < 0.001 in subparts 4–6, *p* = 0.050 in subpart 7) compared to controls. The two CP subgroups did not differ statistically in their MD values.

### Children with CP show structural impairments in the transcallosal fibers

**Fig. 4:**
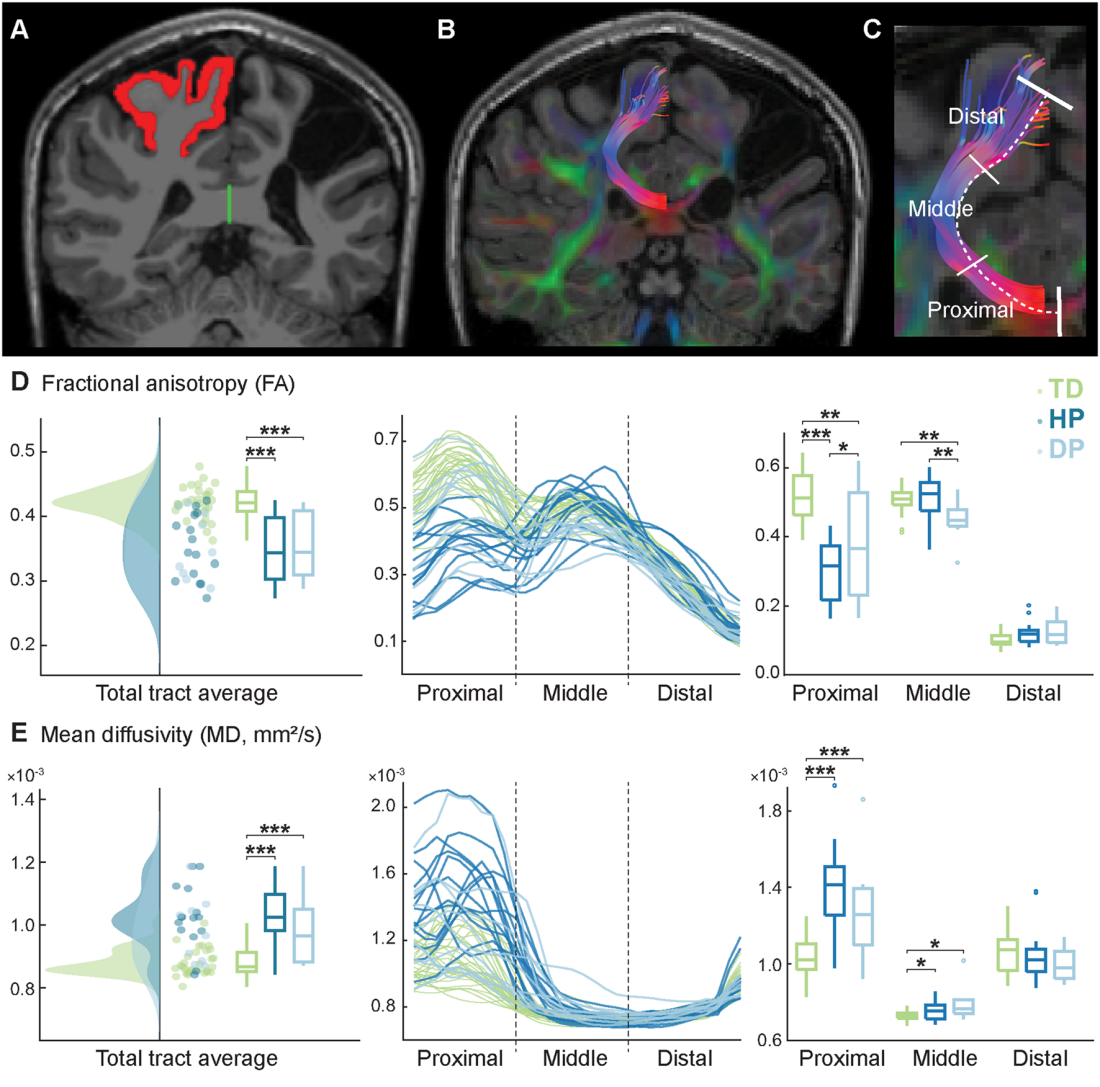
Transcallosal sensorimotor tracts representing the lower limb. **A** For each participant, we created a corpus callosum mask (green) and a cortical mask covering the precentral and postcentral gyri and paracentral lobule (red) for the unaffected or dominant hemisphere. **B** Only tracts connecting these two masks were selected, and additional manual trimming was used to include only the sensorimotor foot tracts. **C** The obtained transcallosal tracts were analyzed in three segments. **D** The whole tract and segment-wise mean FA values. **E** The whole tract and segment-wise mean MD values. *p < 0.05, **p < 0.01, ***p < 0.001

The structural properties of the transcallosal foot tracts differed significantly between children with CP and controls. For the whole tract, both HP and DP groups had a lower number of streamlines, lower average FA and higher average MD (*p* < 0.001 for all pairwise comparisons) compared to controls. The two CP subgroups showed no statistically significant differences for the whole tract. Figure 4D–E shows the FA and MD values for the whole tract (left) and its three segments (right) as well as the values along the tract (middle). It can be noted that the variance between the participants in both FA and MD values was higher close to the midline and lower when moving towards the cortex.

Significant differences between the groups were found in the proximal and middle tract segments but not in the distal segment (i.e., closest to the cortex). In the proximal segment, participants with CP showed decreased FA values (*p* < 0.001 for HP and *p* = 0.001 for DP) and increased MD values (*p* < 0.001 for both HP and DP) compared to controls. Further, the anisotropy in the proximal segment was lower for HP group compared to DP group (*p* = 0.023). In the middle segment, the MD values were increased for both HP and DP groups (*p* = 0.050 for HP and *p* = 0.015 for DP) compared to controls. The middle segment FA values were decreased for DP group when compared to both controls and HP group (*p* = 0.004 for both comparisons). Children with HP did not differ from controls in the middle segment FA values (*p* = 0.45).

The average transcallosal foot tract locations on the CC cross-section are illustrated in Supplementary Figure S2. For DP group, the average locations on the CC cross-section were on subpart 5 (*n* = 5), subpart 6 (*n* = 4) and subpart 7 (*n* = 1), representing the posterior body, isthmus and splenium. For HP group, the locations were either on subpart 5 (*n* = 7) or on subpart 6 (*n* = 8). For TD group, most of the tract locations were on subpart 6 (*n* = 22) and the rest on subpart 5 (*n* = 8). The distribution to different subparts did not differ between the three groups (*p* = 0.10).

### Motor performance is associated with transcallosal white matter properties

Both the multiple linear regression model and Spearman’s partial correlation analyses showed associations between the stability performance and the CC structural properties. After excluding participants with any missing data, the final number of participants in the structure−function analysis was 48 (26 TD, 15 HP and 7 DP). Supplementary Table S1 shows the participant-specific data availability.

#### The multiple linear regression model showed significant associations for static stability

The independent variables of the regression model (group, age, sex, size/FA/MD values of CC subparts and FA/MD values of transcallosal tract segments) did not predict the dynamic stability performance (*p* = 0.39, adjusted* R*^2^ = 0.09). For the static stability, however, the model was statistically significant (*p* < 0.001, adjusted* R*^2^ = 0.73). The model included nine statistically significant coefficients: age; area of CC subpart 4, FA values of CC subparts 2, 5 and 7; MD values of CC subparts 2 and 7; and FA values of proximal and middle transcallosal tract segments. The estimates were positive for subpart 4 area and tract segment FA values, and negative for all other significant variables. For detailed information of the significant coefficients in the model, please see Supplementary Table S2.

#### Correlations were found between diffusivity and dynamic stability in controls

Table 1 shows the rho values of the partial correlation analysis. The only statistically significant (*p* < 0.0018, Bonferroni corrected) correlations were found in the TD group. The dynamic stability of the controls correlated negatively with the MD value of CC subpart 5 (rho = − 0.67, *p* < 0.001) and with the MD value of the proximal segment of the transcallosal tract (rho = − 0.65, *p* < 0.001). A negative rho value indicates that the smaller the diffusion magnitude in this area, the bigger the sway, i.e., worse static stability. Negative correlations were found also in the body of the CC (*p* = 0.008 for subparts 3 and *p* = 0.002 for subpart 4) of the control group although they did not survive Bonferroni correction. For CP groups, the MD of the same CC areas appeared to be, in contrast, positively connected with stability measures but no significant correlations were observed.

**Table 1:** Partial correlations between structural brain measures and stability in typically developed (TD) controls and in adolescents with hemiplegic (HP) or diplegic (DP) cerebral palsy

	TD		HP		DP	
	Static	Dynamic	Static	Dynamic	Static	Dynamic
*Corpus callosum subparts*						
*Area*						
1	0.07	− 0.31	0.40	0.17	0.17	− 0.34
2	0.27	0.19	− 0.07	0.25	− 0.41	− 0.04
3	− 0.02	− 0.04	− 0.11	− 0.15	− 0.47	− 0.51
4	0.16	0.19	− 0.06	− 0.08	− 0.17	− 0.78*
5	0.01	0.28	− 0.17	− 0.14	− 0.10	− 0.53
6	0.07	0.32	0.10	− 0.10	− 0.45	− 0.69
7	− 0.29	− 0.05	0.30	0.06	0.03	− 0.71*
*Fractional anisotropy (FA)*						
1	0.29	0.00	0.28	0.06	− 0.04	− 0.47
2	− 0.17	− 0.05	− 0.11	− 0.38	− 0.68	− 0.37
3	0.00	0.30	− 0.21	− 0.41	− 0.76	− 0.54
4	0.07	0.29	− 0.11	− 0.33	− 0.52	− 0.49
5	0.01	0.41*	− 0.33	− 0.21	− 0.52	− 0.60
6	0.10	0.29	− 0.24	0.00	− 0.82*	− 0.51
7	− 0.08	0.10	− 0.29	− 0.04	− 0.11	− 0.49
*Mean diffusivity (MD)*						
1	− 0.12	− 0.18	− 0.07	− 0.06	0.58	− 0.33
2	0.02	0.12	− 0.34	− 0.14	− 0.41	− 0.23
3	− 0.18	− 0.52*	0.25	0.46	0.57	0.25
4	− 0.10	− 0.59*	0.48	0.39	0.61	0.36
5	− 0.08	− 0.67**	0.38	0.20	0.11	0.39
6	− 0.14	− 0.37	0.01	− 0.15	0.49	0.39
7	− 0.19	0.00	0.09	0.09	− 0.08	0.21
**Transcallosal foot tract segments**						
*Fractional anisotropy (FA)*						
proximal	0.04	0.55*	0.04	− 0.10	− 0.57	− 0.41
medial	0.16	− 0.20	0.13	− 0.08	− 0.35	− 0.65
distal	0.26	0.36	0.30	0.14	0.40	0.25
*Mean diffusivity (MD)*						
proximal	− 0.04	− 0.65**	− 0.15	0.00	− 0.03	0.19
medial	− 0.02	0.23	− 0.18	0.02	0.30	0.63
distal	0.01	− 0.23	− 0.43	− 0.25	− 0.67	− 0.54

*p < 0.05; **p < 0.0018

The remaining variables, subpart size and FA, showed negative correlations with the motor performance in DP group (*p* = 0.023 for size of subpart 4 and *p* = 0.050 for 7, *p* = 0.047 for FA of subpart 6). In contrast, control group showed a positive correlation in FA of subpart 5 (*p* = 0.041). However, none of these correlations survived the multiple comparison correction.

## Discussion

This study extends the previous knowledge on the role of interhemispheric WM structure in CP and its association with motor stability performance. As hypothesized, children with CP showed impairments in the CC size and WM organization as well as in motor stability performance. Children with diplegic CP had less reduced CC cross-sectional area but their transcallosal sensorimotor tracts appeared to be more extensively impaired compared to hemiplegic CP. Finally, we found correlations between the interhemispheric WM structural properties and the motor stability performance both in adolescents with CP and in typically developed controls.

### Children with CP show structural callosal and functional motor impairments

#### Corpus callosum

In line with previous studies on the WM structure of the CC, children with CP showed reduced fractional anisotropy (FA) (Lee et al. 2011; Weinstein et al. 2014; Arrigoni et al. 2016) and increased mean diffusivity (MD) values (Yoshida et al. 2011; Weinstein et al. 2014). Anisotropy and diffusivity are susceptible to a variety of microstructural features including axonal density and diameter, myelination, number of axons and their consistency (Beaulieu 2002). Reduced FA and increased MD in CP might therefore reflect less coherent axonal fiber bundels, thin myelination and/or lower fiber density, possibly resulting in a slower and less dense transmission of interhemispheric information.

Following our hypothesis, the WM impairments were prominent across the body and splenium of the CC. The involvement of the posterior CC subparts is consistent with earlier findings in spastic CP (Lee et al. 2011; Scheck et al. 2012; Arrigoni et al. 2016). The fibers connecting the primary motor cortices typically pass through the body of the CC, whereas fibers from the primary sensory and posterior parietal cortices cross more posteriorly also at the isthmus (Chao et al. 2009; Catani and Thiebaut de Schotten 2012). Therefore, it is logical to see structural WM impairments in these CC areas in children with CP, who experience sensorimotor dysfunction. Hemiplegic and diplegic CP subgroups did not differ in their CC diffusion properties.

Several reports on CC macrostructure have observed a reduced total CC area in both hemiplegic (Hawe et al. 2013) and diplegic CP (Hayakawa et al. 1996; Kułak et al. 2007). However, in our study, the total CC area was reduced only in HP group, whereas no difference was found between DP group and controls. The reduced area in HP group was visible across the body and splenium, while DP group showed a reduced area only in the isthmus. Surprisingly, the comparison between the two patient subgroups revealed significantly reduced areas of the total CC and isthmus in hemiplegic compared to diplegic participants. While the isthmus has been linked to motor functioning (Lee et al. 2011), it is possible that this observation also reflects some behavioral differences between these subgroups. To our knowledge, this is the first time the CC areas of children with hemiplegic or diplegic CP have been compared. Although the observed difference between the hemiplegic and diplegic CP types requires confirmation, it supports the findings of Pagnozzi et al. (2020) who suggested that individuals with different CP subtypes might be differently impaired in their sensorimotor interhemispheric connections. They observed that hemiplegic children with bilateral brain injury have a reduced splenium volume compared to those with unilateral injury (Pagnozzi et al. 2020). These results highlight the need for addressing the heterogeneity of CP also in future research.

#### Transcallosal foot tracts

In both CP subgroups, the transcallosal sensorimotor tract for the lower limbs showed a reduced number of streamlines, decreased total FA, and increased total MD, which align with the results from the CC cross-section analysis. These WM alterations may reflect less coherent interhemispheric connections, suggesting that interhemispheric sensorimotor processing might also be impaired and could thus partly explain the motor impairments in CP. Although no previous study in CP has focused explicitly on the transcallosal foot tracts, these results are consistent with those obtained from other interhemispheric tracts. For example, Weinstein et al. (2014) reported a reduced number of transcallosal streamlines across the CC, and lower anisotropy and higher mean diffusivity in transcallosal tracts of the CC midbody in children with hemiplegic CP. Furthermore, reduced FA values have been reported in spastic CP for transcallosal motor tract (Koerte et al. 2011) and tracts passing through the genu and splenium (Jiang et al. 2019), and in dyskinetic CP for transcallosal sensorimotor tracts (Ballester-Plané et al. 2017). In a recent study by Papadelis et al. (2019), children with hemiplegic CP had increased MD values but no difference in FA values when analyzing all fibers travelling through CC. To our knowledge, this is the first time the altered WM structure of the transcallosal sensorimotor foot tracts has been presented in participants with spastic CP.

Although several authors have utilized tractography to quantify transcallosal connections, reporting the along-tract WM structure is scarce. By extending the research on along-tract analysis, our study showed that the structural impairments on the transcallosal foot tracts were most prominent in the proximity of the brain midline, especially in participants with hemiplegic CP. One explanation for this result could be that diffusion metrics, especially FA, are known to be more robust in regions without crossing fibers or fanning such as the CC at the midline (Tournier et al. 2011). In a transcallosal tract study in infants with bilateral CP by Jiang et al. (2019), in contrast, no such along-tract spatial dependency of the impairments was found. The FA values of tracts passing through genu and splenium seemed to be similarly reduced along their course, which could be explained by the exclusion of cortical tract segments in their analysis (Jiang et al. 2019).

Our results further demonstrated a significant difference between hemiplegic and diplegic CP subgroups in the FA values of the transcallosal foot tracts. HP group showed significantly lower FA values closer to the brain’s midline, whereas DP group had decreased FA in the middle segment of the tract. The deviant FA trajectories of the transcallosal foot tracts could indicate that the myelinization or coherency of the transcallosal foot tract is differently altered in the two patient groups. This variation between the hemiplegic and diplegic children was not detectable at the whole-tract-level. Therefore, we highlight the importance of along-tract analysis or novel methods such as fixel-based analysis (Dhollander et al. 2021) in future. It is noteworthy that we decided to include only the tracts of the unaffected hemisphere in the current analysis to achieve robust and reproducible tractography results. In the presence of gross malformations or lesions, the extraction and meaningful comparison of the tracts in the affected hemisphere was discovered challenging and imprecise. However, children with hemiplegic CP typically have a unilateral brain injury and thus the transcallosal tract in the unaffected hemisphere can have less impaired microstructure than the tract in the affected hemisphere would. To develop a full picture of the transcallosal tract involvement in these two CP subtypes, even more detailed studies will be needed that also include the affected hemisphere in the analyses.

#### Static and dynamic stability

Based on previous analyses of primarily overlapping dataset (Piitulainen et al. 2021), we expected the children with CP to have poorer overall static and dynamic stability compared to controls. Piitulainen et al. (2021) demonstrated that HP patients had a more stable gait than DP in the vertical direction of the acceleration predominantly during the cognitive dual task, but not in the horizontal direction. In the current report, we combined vertical and horizontal directions of all tasks in the dynamic stability sum variable, and thus did not find a statistical difference between CP subgroups. We chose to use this sum variable to obtain a comprehensive estimate for the dynamic stability in the current study. Furthermore, Piitulainen et al. suggested that the difficulties experienced in static stability may transfer to dynamic stability in children with or without CP and indeed showed that the dynamic and static stability were correlated (Piitulainen et al. 2021). However, in the current study, we found it important to study the static and dynamic stability performance separately as they might still be individually impaired in the heterogenous CP population.

### Associations between motor stability performance and transcallosal white matter properties

We found significant associations between the motor stability performance and the structural properties of the CC and transcallosal foot tracts. The linear regression analysis showed a significant relationship between the WM structural properties and static standing stability, but not with the dynamic gait stability. Correlation analyses, in contrast, were significant only between the structural variables and dynamic stability in the control group. Correlations within the two CP subgroups did not survive the multiple comparison correction, which can be the result of the smaller number of the patients compared to controls.

These associations between the WM structural properties and motor stability performance were found both in the CC cross-section and transcallosal foot tracts. The static standing stability was associated with the genu, the middle and posterior body, and the splenium of CC. These areas accord with the findings in the aging population, where the same regions have been related to static stability (Sullivan et al. 2010; Brodoefel et al. 2013). In contrast, studies in younger adults (van Impe et al. 2012) and in adolescents (Groeschel et al. 2019) have failed to find an association between the CC structure and static stability. To our knowledge, this is the first time the relationship between CC structure and static stability has been demonstrated in an adolescent population and in CP.

Dynamic gait stability was associated with the WM structural properties of the posterior body of CC and the transcallosal foot tract in controls. Previous studies have linked the gait performance with the structural properties of anterior CC (Meyns et al. 2016; Papageorgiou et al. 2020), but not with the more posterior sensorimotor parts of the CC. We also showed significant association between the dynamic stability and transcallosal sensorimotor tracts, detected only in the most proximal third of the tract with respect to the midline. These results reflect those of Grohs et al. (2018) in typically developed preschoolers, who reported the relationship between motor performance (including static and dynamic stability) and transcallosal motor tracts to be prominent in the medial part of the tract.

Typically, similar structure−function studies have shown that smaller CC size and lower WM integrity (i.e., lower FA and higher MD) are correlated with poorer motor performance (Lee et al. 2011; Groeschel et al. 2019; Hung et al. 2019; Robert et al. 2021). On the contrary in the present study, the directionality of the structure−function relationship was non-conclusive, and especially the correlation directions in the control group contradicted our hypotheses. Unexpected correlation directions between the motor performance and CC structure have been also previously reported in young adults and a finger-tapping task (Fling et al. 2011), and in young adults, born preterm and their gross motor function (Hollund et al. 2018). These findings indicate that the CC structure–motor function association appears to be complex and may be manifested differently in different populations. To date, it is not fully understood whether the role of CC in the sensorimotor network is inhibitory, facilitating brain lateralization, or excitatory, sharing of information between the hemispheres (for a review, see van der Knaap and van der Ham 2011). Furthermore, the interpretation of FA and MD metrics at a macrostructural level remains challenging (Tournier et al. 2011; Jones et al. 2013). Thus, it is not straightforward to predict how the structural measures of the interhemispheric WM pathways should be related to the functional motor performance. In addition, our biomechanical motor performance metrics reflected overall motor stability during bilateral tasks but did not separate the limb or side-specific aspects of the motor performance. Therefore, further studies could aim to quantify the limb-specific motor performance to reveal possible associations between the transcallosal pathways and the side-dependent motor performance, that may be evident especially in hemiplegic CP.

### Limitations

In the present study, we analyzed the structural properties of CC at a single midsagittal slice. Although the manual outlining of CC cross-section was found the most accurate and consistent method in the current dataset, future studies could benefit from volumetric segmentation of the corpus callosum and verification of more than one expert. Furthermore, the risk of partial volume effects cannot be ignored, as the CC size might influence the measured diffusion properties. This is the case especially in smaller parcels, such as rostrum, where the number of voxels was low (range: 9–49 voxels) and thus the risk of partial volume effects influencing the result is high and the statistical power may be hindered. The tractography analysis on the other hand was limited to craniocaudal transcallosal sensorimotor tracts, referred to as “transcallosal foot tracts” in this paper. The confirmation of the true representative foot locations in the sensorimotor cortex would, however, require functional imaging such as transcranial magnetic stimulation or functional MRI.

Although the number of participants in this study was satisfactory, the number of complete datasets was lower especially in the DP group. Thus, the correlation analyses would require confirmation with larger patient groups, which could also make it possible to include more background information, such as gestational week and type of the brain lesion. Another limitation of the study is that sex was left uncontrolled, even though it might induce some biases (Luders et al. 2010). We also acknowledge that the 8-year age range (10 − 18) of the participants might add some developmental influence on the results despite we controlled for age in our correlation analyses.

Finally, we note that our dMRI analysis was limited to the standard diffusion tensor model indices, i.e., FA and MD. The used imaging protocol with *b* = 1000 does not, however, necessarily comply as well with modern biophysical models, such as NODDI (Zhang et al. 2012). For the same reason, more sophisticated, microstructure-informed tractography methods (Sairanen et al. 2022) are not relevant for the current dataset but might benefit future studies. Although the analysis did not include CSD-based metrics such as apparent fibre density (AFD; Raffelt et al. 2012) and hindrance modulated orientational anisotropy (HMOA; Dell’Acqua et al. 2013), we observed that these values appeared to support the findings obtained with tensor-based metrics.

## Conclusions

Firstly, this study identified significant structural differences in the CC and in the transcallosal sensorimotor tract between adolescents with CP and their healthy peers, and between hemiplegic and diplegic CP subgroups. These results indicate the significance of the interhemispheric WM structures in the attempt to understand the brain mechanisms in spastic CP and may help to further develop and evaluate specific rehabilitation processes. Secondly, associations were found between interhemispheric WM structural properties and motor stability performance. Underscoring the complexity of the structure−function relationship, the found associations failed to show any conclusive directionality. In conclusion, this study offers new insights into the complicated interplay between the brain’s WM organization and motor stability performance in children with CP and their typically developed peers.

### Supplementary Information

Below is the link to the electronic supplementary material.

Supplementary file1 (DOCX 1188 KB)

## Data Availability

The data that support the findings of this study are available upon reasonable request from the corresponding author. The data are not publicly available due to privacy or ethical restrictions.

## Abbreviations

CC: Corpus callosum
CP: Cerebral palsy
CSD: Constrained spherical deconvolution
dMRI: Diffusion-weighted MRI
DP: Diplegic cerebral palsy
FA: Fractional anisotropy
FOD: Fiber orientation distribution
FSIQ: Full-scale intelligence quotient
GMFCS: Gross motor functioning classification scale
HP: Hemiplegic cerebral palsy
MD: Mean diffusivity
TD: Typically developed
WM: White matter
