# Supplementary material for: Altered corpus callosum structure in adolescents with cerebral palsy: connection to gait and balance
Source: Brain Struct Funct. 2023 Aug 24;228(8):1901–15. doi: 10.1007/s00429-023-02692-1 (PMC10516810; doi:10.1007/s00429-023-02692-1)
Supplement: Supplementary file 1 — Supplementary file1 (DOCX 1188 KB) [file 429_2023_2692_MOESM1_ESM.docx]

**Supplementary Table S1 Participant demographics and data availability**

| **ID** | **Group** | **Age** | **Sex** | **Dominant hand** | **Stability** | **MRI** | **Lesion type** |
| --- | --- | --- | --- | --- | --- | --- | --- |
| 1 | TD | 10 | Female | Right | Yes | Yes | – |
| 2 | TD | 10 | Female | Right | Yes | Yes | – |
| 3 | TD | 11 | Male | Right | Yes | Yes | – |
| 4 | TD | 11 | Female | Right | Yes | Yes | – |
| 5 | TD | 12 | Female | Right | Yes | Yes | – |
| 6 | TD | 12 | Male | Right | Yes | Yes | – |
| 7 | TD | 12 | Male | Right | Yes | Yes | – |
| 8 | TD | 12 | Male | Right | Yes | No | – |
| 9 | TD | 12 | Male | Right | Yes | Yes | – |
| 10 | TD | 12 | Male | Right | Yes | No | – |
| 11 | TD | 12 | Male | Right | Yes^a^ | Yes | – |
| 12 | TD | 12 | Female | Right | Yes | Yes | – |
| 13 | TD | 12 | Female | Left | Yes | Yes | – |
| 14 | TD | 12 | Female | Right | Yes | Yes | – |
| 15 | TD | 13 | Male | Right | Yes | Yes | – |
| 16 | TD | 13 | Male | Right | Yes | Yes | – |
| 17 | TD | 13 | Female | Right | Yes | Yes | – |
| 18 | TD | 13 | Male | Right | Yes | No | – |
| 19 | TD | 14 | Male | Right | Yes | Yes | – |
| 20 | TD | 14 | Female | Right | Yes | No | – |
| 21 | TD | 14 | Male | Right | Yes | Yes | – |
| 22 | TD | 14 | Female | Right | Yes | Yes | – |
| 23 | TD | 14 | Female | Right | Yes | Yes | – |
| 24 | TD | 14 | Female | Right | Yes | No | – |
| 25 | TD | 16 | Female | Right | Yes | Yes | – |
| 26 | TD | 16 | Female | Right | Yes | Yes | – |
| 27 | TD | 16 | Female | Right | Yes | Yes | – |
| 28 | TD | 17 | Female | Right | Yes | Yes | – |
| 29 | TD | 17 | Female | Right | Yes | Yes | – |
| 30 | TD | 17 | Male | Right | No | Yes | – |
| 31 | TD | 17 | Female | Right | Yes | Yes | – |
| 32 | TD | 17 | Male | Left | Yes | Yes | – |
| 33 | TD | 18 | Male | Right | Yes | Yes | – |
| 34 | TD | 18 | Female | Right | No | Yes | – |
| 35 | TD | 18 | Female | Right | No | Yes | – |
| 36 | HP | 10 | Female | Left | Yes | Yes | Infarction |
| 37 | HP | 11 | Male | Left | Yes | Yes^c^ | lHI |
| 38 | HP | 11 | Female | Right | Yes | Yes | Asphyxia |
| 39 | HP | 11 | Female | Right | Yes | Yes | Infarction |
| 40 | HP | 11 | Female | Left | Yes | Yes | lHI, mild PVL |
| 41 | HP | 12 | Male | Left | Yes | No | lHI |
| 42 | HP | 12 | Male | Left | Yes | Yes | Infarction |
| 43 | HP | 13 | Female | Right | Yes | Yes | PVH/Infarction, lHI, PVL |
| 44 | HP | 13 | Male | Left | Yes | Yes | Infarction |
| 45 | HP | 13 | Female | Left | Yes | Yes | Infarction |
| 46 | HP | 14 | Male | Right | Yes | Yes | IVH, PVL |
| 47 | HP | 14 | Female | Right | Yes | Yes | perinatal injury |
| 48 | HP | 14 | Female | Left | Yes | Yes | PVL |
| 49 | HP | 15 | Male | Left | Yes | Yes | lHI, IVH, WMI, PVL, WD |
| 50 | HP | 16 | Female | Left | Yes | No | Infarction |
| 51 | HP | 17 | Female | Right | Yes | Yes | Local infarction/hemorrhage |
| 52 | HP | 17 | Female | Left | Yes | Yes | Infarction |
| 53 | HP | 18 | Female | Left | Yes | Yes | Unknown |
| 54 | DP | 11 | Female | Right | Yes | No | HIBI |
| 55 | DP | 11 | Female | Right | Yes | Yes | PVL |
| 56 | DP | 11 | Male | Right | Yes | Yes^c^ | PVL, local ischemia |
| 57 | DP | 11 | Female | Right | Yes^b^ | Yes | HIE, ICH, PVL |
| 58 | DP | 11 | Male | Left | Yes | Yes | PVL |
| 59 | DP | 12 | Male | Left | Yes | Yes | HIBI, PVL |
| 60 | DP | 13 | Female | Right | Yes | Yes | PVL, infection |
| 61 | DP | 14 | Male | Left | No | Yes | ICH, IVH, hemorrhagic infarction |
| 62 | DP | 14 | Male | Right | Yes | No | HIBI |
| 63 | DP | 15 | Male | Right | Yes^b^ | Yes | Unknown |
| 64 | DP | 15 | Female | Right | Yes | Yes | Unknown |
| 65 | DP | 15 | Female | Right | Yes | Yes | MRI normal |
| 66 | DP | 17 | Male | Left | Yes | Yes | Unknown |

Table shows participant demographics including group (TD, typically developed controls; HP, hemiplegic cerebral palsy; DP, diplegic cerebral palsy), age, sex and handedness. Both stability assessment and MRI session were completed successfully from most of the participants with few exceptions:

^a^ no static stability data

^b^ no dynamic stability data

^c^ no diffusion-weighted MRI data

Lesion type of the patients was determined by a clinician at the time of diagnosis. Abbreviations: PVL, periventricular leukomalacia; PVH, periventricular hemorrhage; HIBI, hypoxic-ischemic brain injury; lHI, local hypoxia–ischemia; WMI, white matter injury; WD, Wallerian degeneration; IVH, intraventricular hemorrhage; ICH, intracerebral hemorrhage.

**Supplementary Table S2 Statistically significant (p < 0.05) coefficients of the static stability multiple linear regression model.**

| Coefficient | Estimate | Std.error | t-value | p-value |
| --- | --- | --- | --- | --- |
| Age | -0.032 | 0.010 | -3.146 | 0.006 |
| Area of CC subpart 4 | 0.009 | 0.004 | 2.344 | 0.031 |
| FA of CC subpart 2 | -3.822 | 1.054 | -3.627 | 0.002 |
| FA of CC subpart 5 | -1.875 | 0.783 | -2.394 | 0.028 |
| FA of CC subpart 7 | -1.822 | 0.541 | -3.367 | 0.004 |
| MD of CC subpart 2 | -648.8 | 299.2 | -2.168 | 0.045 |
| MD of CC subpart 7 | -1248.0 | 359.2 | -3.474 | 0.003 |
| FA of tract segment 1 | 1.954 | 0.506 | 3.858 | 0.001 |
| FA of tract segment 2 | 1.216 | 0.530 | 2.295 | 0.035 |


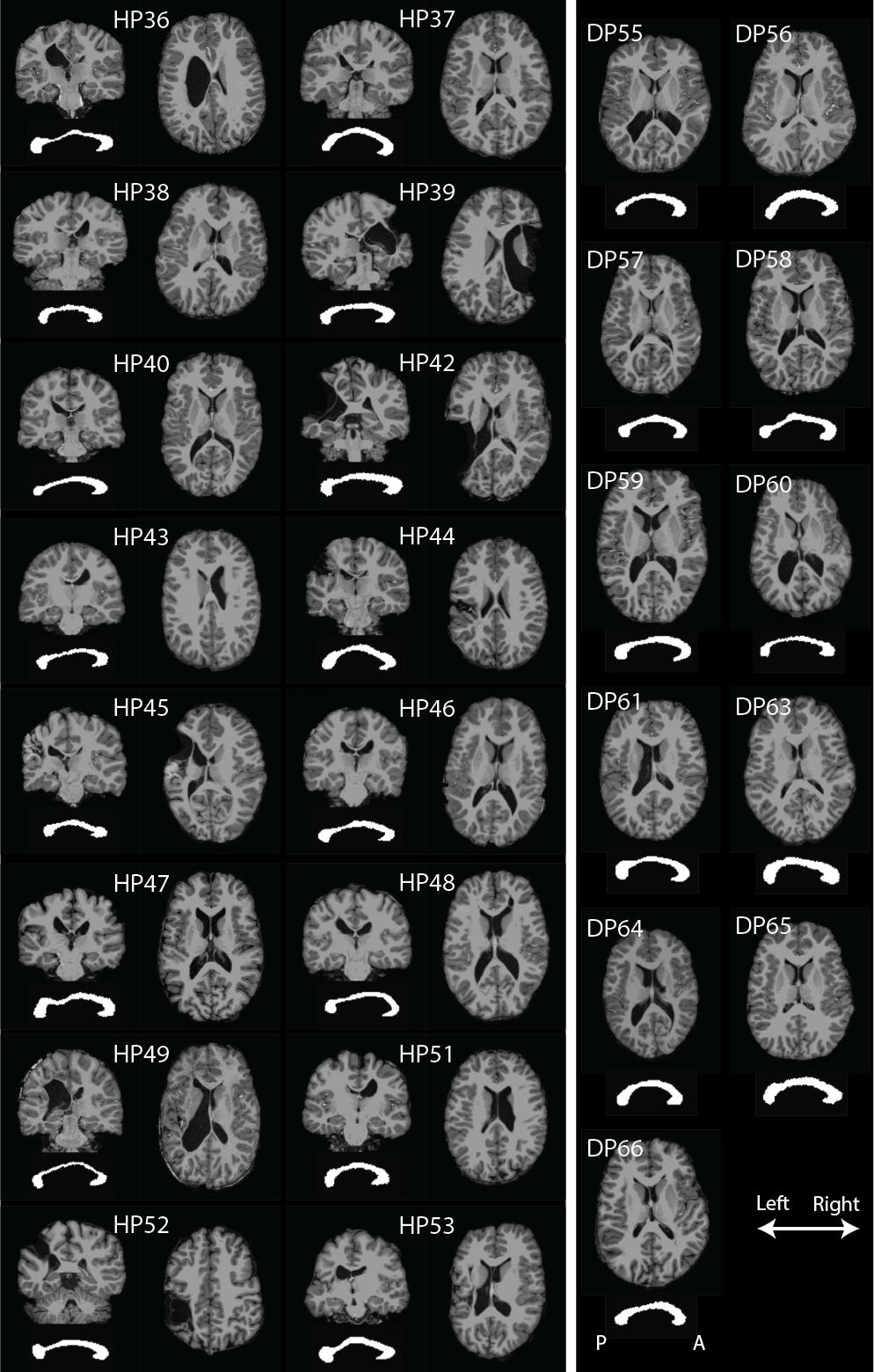
**Supplementary Fig. S1** **Patient MRIs**. HP, hemiplegic cerebral palsy; DP, diplegic cerebral palsy


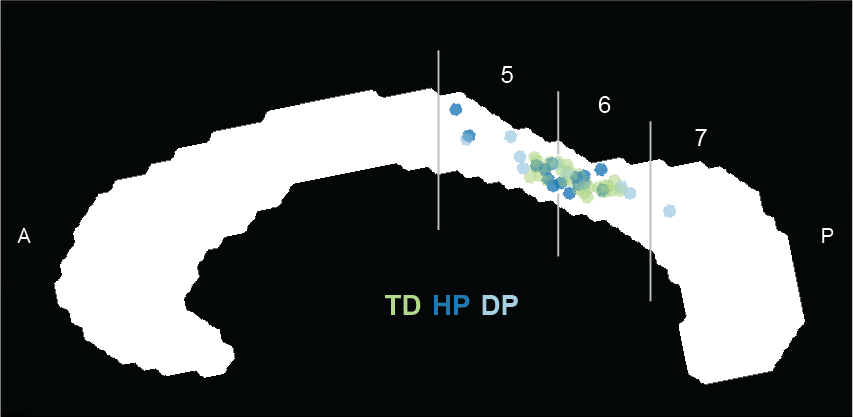


**Supplementary Fig. S2 Average transcallosal sensorimotor foot tract locations on corpus callosum**. TD, typically developed control; HP, hemiplegic cerebral palsy; DP, diplegic cerebral palsy
